# The new insight into the role of hydroxyproline in metabolism of cancer cells

**DOI:** 10.3389/fcell.2025.1556770

**Published:** 2025-05-16

**Authors:** Magda Chalecka, Justyna Magdalena Hermanowicz, Jerzy Palka, Arkadiusz Surazynski

**Affiliations:** ^1^ Department of Medicinal Chemistry, Medical University of Bialystok, Bialystok, Poland; ^2^ Department of Pharmacodynamics, Medical University of Bialystok, Bialystok, Poland

**Keywords:** hydroxyproline, prolidase, collagen, OH-POX/PRODH2, HIF-1α, cancer cells

## Abstract

Although the role of proline (Pro) in regulatory mechanisms of cell metabolism is well recognized, the interest in metabolic significance of hydroxyproline (Hyp) has received little attention. Hyp was considered as a waste metabolite of protein degradation, mainly degradation of collagen. This amino acid is not synthesized *de novo* and is not incorporated into proteins. Hyp is a product of Pro hydroxylation in proteins by specific Pro hydroxylase. Therefore, Hyp is derived from degradation of proteins, and it is further metabolized by specific Hyp dehydrogenase 2 (PRODH2), known also as a Hyp oxidase (OH-POX). The enzyme catalyzes conversion of Hyp into Δ^1^-pyrroline-3-OH-5-carboxylic acid (OH-P5C), yielding electrons that are used in electron transport chain for ATP production. However, in certain conditions the electrons are accepted by oxygen forming reactive oxygen species (ROS). The product, OH-P5C could be also converted by OH-P5C reductase to recycle NADP^+^ in pentose phosphate pathway (PPP), yielding nucleotides for DNA synthesis. Interestingly, Pro hydroxylase requires the same cofactors (α-KG, ascorbate and Fe^2+^) as a DNA and histone demethylases, suggesting the role of Pro hydroxylation in epigenetic regulation. Hyp could be also converted to highly energetic amino acid, glycine (Gly). Of great importance is the role of Hyp in upregulation of transcriptional activity of hypoxia-inducible factor 1α (HIF-1α), inducing angiogenesis and metastasis. Therefore, Hyp is involved in several critical metabolic processes regulating DNA synthesis, gene expression, apoptosis/survival, angiogenesis, metastasis and energy production, suggesting a key role of Hyp in reprogramming metabolism of cancer cells. It suggests that Hyp metabolism could be considered as a target in novel experimental strategy for cancer treatment.

## 1 Introduction to Hyp metabolism

Several lines of evidence suggest that non-essential amino acids (NEAAs), particularly proline (Pro) and hydroxyproline (Hyp) play a key role in regulation of cellular metabolism (Phang, 2022; Tanner et al., 2018; Wu et al., 2019; Karna et al., 2020; Geng et al., 2021). Although the role of Pro in complex regulatory mechanisms of cell metabolism is well established (Phang, 2019; D'Aniello et al., 2020; Geng et al., 2021), the significance of Hyp in these processes has attracted less attention. However, it is a unique amino acid. Hyp is not synthesized *de novo*. Its formation in the cells is a result of post-translational hydroxylation of Pro in proteins, particularly in the chains of newly synthesized collagen subunits (Gorres and Raines, 2010). Hyp and Pro represent 12.5% (g/g) of mammal’s proteins. The ratio of both amino acids is 1:2.25 (Hyp:Pro). Collagen contains 13.0 Pro and 9.1 Hyp residues per 100 amino acid residues (13.30 g of Pro residues and 10.84 g of Hyp residues per 100 g of collagen) (Wu et al., 2019). Therefore, the presence of free Hyp in the cells result mainly from collagen degradation. However, the source of endogenous Hyp is not only collagen. Hyp is also present in a large amount in elastin, C1q component of the complement system, acetylcholinesterase or ectodiplasmin A (Bochicchio et al., 2013; Srivastava et al., 2016).

**FIGURE 1:**
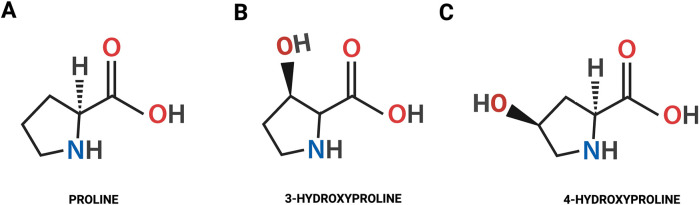
The chemical structure of the proline **(A)** and the isomeric forms of hydroxyproline: trans-4-hydroxy-L-proline **(B)**; trans-3-hydroxy-L-proline **(C)**.

Hydroxylation of Pro plays a critical role in collagen biosynthesis and transport of the protein to the extracellular matrix (ECM). The hydroxylation of Pro to Hyp occurs in the endoplasmic reticulum (ER) (Myllyharju, 2005). In mammals Hyp exists in the L-configuration in the form of 4-hydroxy-L-proline (4-Hyp) and 3-hydroxy-L-proline (3-Hyp). In collagen 4-Hyp and 3-Hyp are present only in trans isoforms. Trans-4-Hyp is formed by attachment of hydroxyl group (-OH) to the γ carbon atom, while in trans-3-Hyp the -OH is attached to the β carbon atom (Figure 1) (Gorres and Raines, 2010; Aro et al., 2015; Srivastava et al., 2016).

**FIGURE 2:**
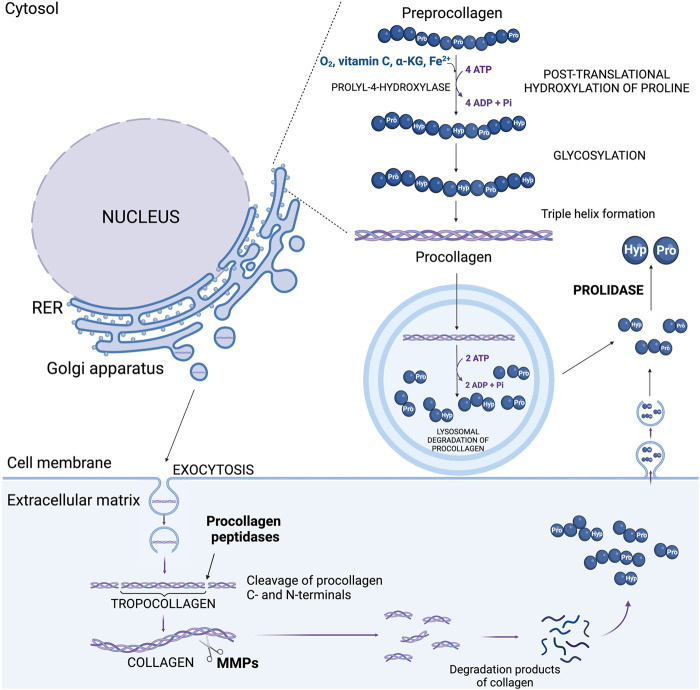
The sources of hydroxyproline in the cell: process of post-translational proline hydroxylation in new collagen and pathway of collagen degradation. ADP, adenosine diphosphate; ATP, adenosine triphosphate; α-KG, α-ketoglutarate; Hyp, hydroxyproline; MMPs, metalloproteinases; Pro, proline; RER, rough endoplasmic reticulum.

Hydroxylation of Pro residues in the collagen polypeptide chains is catalyzed by prolyl-3-hydroxylase (EC 1.14.11.7.) and prolyl-4-hydroxylase (EC 1.14.11.2.) in the presence of α-ketoglutarate (α-KG), ascorbic acid, oxygen, and ferric ions (Fe^2+^) (Myllyharju, 2005; Ishikawa and Bächinger, 2013). Hydroxylases are stereoselective and hydroxylate Pro residues in the Yaa position of the Gly-Xaa-Yaa sequence of collagen chains (Ishikawa and Bächinger, 2013). This modification is necessary for the proper folding of procollagen, the precursor of collagen, and formation of collagen triple helical structure (Ishikawa et al., 2015). Although Hyp does not participate in hydrogen bonding, it promotes the formation of hydrogen bonds between collagen subunits, which increases the thermal stability, mechanical strength and integrity of the triple helix (Shoulders and Raines, 2009; Onursal et al., 2021; Ishikawa et al., 2015). Proper folding is necessary for the transport of procollagen to the Golgi apparatus. Hyp ensures that procollagen molecules achieve a conformation that prevents their intracellular degradation. Once properly folded, procollagen is packaged into vesicles and transported from the ER to the Golgi apparatus (Santos et al., 2015). In the Golgi, further modifications such as glycosylation occur, and procollagen is then secreted into the extracellular space via secretory vesicles (Ishikawa and Bächinger, 2013; Lavieu et al., 2014). The vesicles containing procollagen are transported to the cell surface, where they fuse with the plasma membrane and release procollagen into the extracellular space (Myllyharju, 2005). In the ECM, specific enzymes called procollagen peptidases cleave the N- and C-terminal propeptides from procollagen, converting it into collagen (Figure 2) (Onursal et al., 2021; Robins, 2007). Hyp-rich regions ensure that the collagen molecules remain stable during this process. The mature collagen molecules self-assemble into fibrils, which further organize into fibers. The presence of Hyp is crucial for the stability and proper alignment of these fibrils. Therefore, Hyp indirectly influences the formation of covalent cross-links between collagen molecules catalyzed by lysyl oxidase (Robins, 2007; Vallet and Ricard-Blum, 2019). These cross-links are essential for the tensile strength and structural integrity of the collagen fibers in the ECM. This process is tightly controlled by paracrine and endocrine regulation. Disturbances of this process has functional significance in some ECM diseases, e.g., arthritis (Guszczyn et al., 2009) and cancer (Hulahan and Angel, 2024). Degradation of collagen contributes to release of amino acids, including Hyp. This process is initiated by collagen cleavage in ECM by metalloproteinases (e.g., MMP-2, MMP-9) producing two globular collagen fragments that after internalization are further degraded in lysosomes into amino acids (Malemud, 2006; Theocharis et al., 2019). However, lysosomal enzymes are unable to cleave di- and tri-peptides containing C-terminal Pro or Hyp. These imidopeptides are cleaved by cytosolic imidopeptidase, prolidase (PEPD), releasing Pro, Hyp and other amino acids (Eni-Aganga et al., 2021; Wilk et al., 2021). These processes are augmented at hypoxic condition, inflammation or oxidative stress contributing to increase in intracellular pool of Hyp (Wenger and Hoogewijs, 2010; Chandel, 2010). It is a consequence of collagen degradation that is intensified during inflammation (pro-inflammatory cytokines enhance the activity of MMPs) and tumor progression (Krane, 2008; Srivastava et al., 2016). In such a condition a large amount of tissue collagen in the microenvironment is degraded, thereby contributing to increase in the concentration of Pro and Hyp (Liotta, 1986; Kitchener and Grunden, 2012) that participate in reprogramming of cellular metabolism (Elia et al., 2017). However, Pro can be derived also from glutamine (Glu), ornithine (Orn), arginine (Arg) (Smith and Phang, 1979) and intermediates of tricarboxylic acid cycle (TCA), e.g., α-KG (Chalecka et al., 2021).

Pro and Hyp are metabolized by different pathways and play different roles in the organism. The degradation of these amino acids is catalyzed by mitochondrial enzymes, proline dehydrogenase (PRODH) known also as proline oxidase (POX) and hydroxyproline dehydrogenase (PRODH2), known also as hydroxyproline oxidase (OH-POX), respectively. Pro is used mainly for protein synthesis. However, it is also an important player in transferring redox equivalents between the mitochondrion and cytosol (Hagedorn and Phang, 1983). This process catalyzed by PRODH/POX is involved in regulation of several cellular and energetic processes (Chalecka et al., 2021). Hyp, unlike Pro, is not used for protein synthesis. There is also no conversion of Hyp to Pro (Gorres and Raines, 2010). Hyp is degraded into two- and three-carbon compounds - glyoxylate and pyruvate (Cooper et al., 2008). Released Hyp was found to be involved in the regulation of apoptosis/survival, antioxidant defense, angiogenesis, energy production, tumorigenesis, and epigenetic regulation. These functions of Hyp are described in further sections of the manuscript.

## 2 The role of Hyp in energy production and redox homeostasis

**FIGURE 3:**
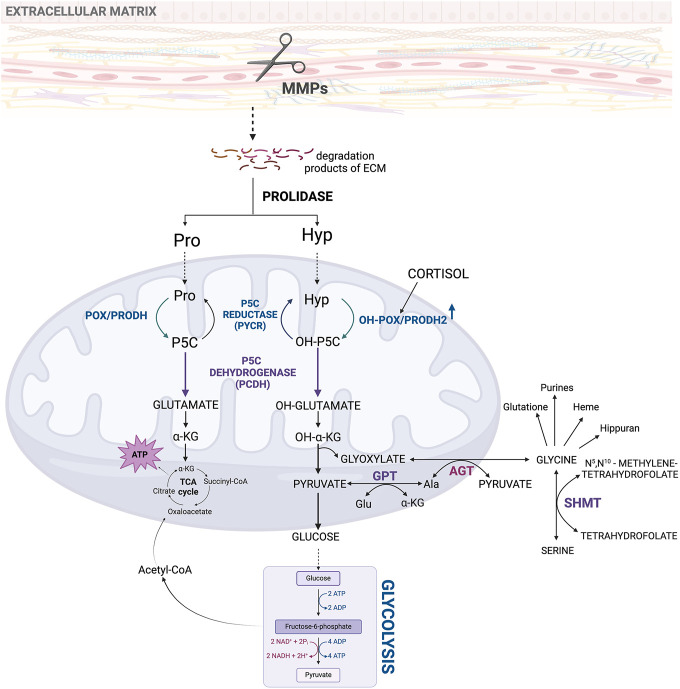
Metabolic pathway of hydroxyproline as an initial product for intracellular interconversions and processes. Acetyl-CoA, acetyl coenzyme A; ADP, adenosine diphosphate; AGT, alanine-glyoxylate aminotransferase; Ala, alanine; ATP, adenosine triphosphate; ECM–extracellular matrix; Glu, glutamate; GPT, glutamate pyruvate transaminase; Hyp, hydroxyproline; MMPs, metalloproteinases; NAD+, oxidized form of nicotinamide adenine dinucleotide; NADH, reduced form of nicotinamide adenine dinucleotide; OH-POX/PRODH2 - hydroxyproline oxidase/proline dehydrogenase 2; OH-P5C, Δ1-pyrroline-3-OH-5-carboxylic acid; OH-α-KG, α-hydroxyketoglutarate; POX/PRODH, proline oxidase/proline dehydrogenase; Pro, proline; P5C, Δ1-pyrroline-5-carboxylate; SHMT, serine hydroxymethyltransferase; TCA cycle, tricarboxylic acid cycle.

The involvement of Hyp in energy production and redox homeostasis is determined by its metabolism. Degradation of Hyp is catalyzed by PRODH2/OH-POX. The enzyme converts Hyp to Δ^1^-pyrroline-3-OH-5-carboxylic acid (OH-P5C) (Adams and Frank, 1980). As previously described, an increase in MMPs activity provides substrates (Pro and Hyp) for PRODH/POX and PRODH2/OH-POX-dependent generation of ATP for survival or reactive oxygen species (ROS) initiating apoptotic cell death (Donald et al., 2001; Kazberuk et al., 2022). Mitochondrial PRODH2/OH-POX is widely distributed in animal tissues; nevertheless, its highest expression is found in the kidney (Lowry et al., 1985). The enzyme activity of PRODH2/OH-POX regulates the conversion rate of Hyp to Gly (Wu et al., 1999). The PRODH2/OH-POX is stimulated by cortisol, suggesting that it is a stress responding enzyme (Figure 3) (Wu et al., 2019; Phang et al., 2015). Cortisol-dependent increase in PRODH2/OH-POX expression results in an increase in Gly synthesis from Hyp. This process has several physiological and therapeutic implications. This pathway plays a key role in collagen metabolism, stress adaptation, wound healing and antioxidant defense. Cortisol-stimulated PRODH2/OH-POX expression contributes also to the increase in glutathione level protecting cells from harmful effects of ROS. Furthermore, degradation of Hyp by PRODH2/OH-POX contributes to increase in α-KG, which supports the TCA cycle to produce ATP. Despite the benefits of increased Gly synthesis (described in more detail in the next chapter), this amino acid could be degraded by enhanced activity of methyl-tetrahydrofolate-dependent Gly cleavage system (GSC) and serine (Ser) tetrahydrofolate-dependent serine hydroxymethyltransferase (SHMT) particularly active in the kidney. This suggests the need for creation of a cortisol analog that, despite stimulating PRODH2/OH-POX expression, would not activate renal GSC and SHMT. Such an analog could selectively upregulate PRODH2/OH-POX expression and convert Hyp to Gly (Wu et al., 2011), without increasing GSC and SHMT activity (Wu et al., 2019). Such an approach would improve cellular metabolic stability by increasing the supply of Gly for collagen biosynthesis, antioxidant defense and wound healing, while preventing degradation of this amino acid in the kidney.

Hyp, similarly as Pro participate in oxidation/reduction (redox) processes in the cell (Phang et al., 2015; Phang and Liu, 2012). Cycling of Pro into P5C catalyzed by PRODH/POX and conversion of P5C to Pro catalyzed by P5C reductase (PYCR) maintains redox homeostasis. The process of shuttling Pro into P5C and back to Pro is called “proline cycle” (Tanner et al., 2018; Phang, 2021; Chalecka et al., 2021; Hu, 2021). Conversion of P5C to Pro transfers oxidative potential that is coupled to pentose phosphate pathway (PPP) producing nucleotides for DNA synthesis (Chalecka et al., 2021). Similarly, conversion of Hyp into OH-P5C by PRODH2/OH-POX and reversal reaction catalyzed by OH-P5C reductase ensures redox balance (Valle et al., 1979). This transformation can be named by analogy to the proline cycle, “hydroxyproline cycle”. Similarly, “hydroxyproline cycle” through PPP contributes to maintain substrates for DNA synthesis. Deregulation of the balance could be induced by cortisol, steroid that upregulate PRODH2/OH-POX expression (Phang et al., 2010).

Moreover, Hyp has potential activity as an antioxidant scavenging ROS (Phang and Liu, 2012; Phang et al., 2015; Hu et al., 2022). It has been documented that Hyp inhibits hydroxyl radical formation in the Fenton reaction by formation of coordination bonds with iron, sequestration of Fe^2+^ and interaction with intermediates through hydrophobic hydration (Milić et al., 2015). This function of Hyp could explain the phenomenon of increased Hyp concentration in the cell due to collagen degradation in response to oxidative stress and hypoxia (Phang and Liu, 2012; Chandel, 2010; Wu et al., 2019). Furthermore, Hyp as a substrate for the synthesis of Gly supports synthesis of DNA, and several other substances as heme, glutathione, serine, purines or hippuran (Figure 3) (Wang et al., 2014).

PRODH/POX and PRODH2/OH-POX are encoded by two different genes, *PRODH1* and *PRODH2*, respectively. Although, there is a slight overlap of the enzyme specificity toward OH-P5C and P5C, the end products of these two degradation pathways are α-KG for Pro and glyoxylate and pyruvate for Hyp (Hu et al., 2022; Valle et al., 1979; Adams and Goldstone, 1960). However, both P5C and OH-P5C can be transformed by PYCR and P5C dehydrogenase (P5CDH) forming Pro and Glu, respectively (Cooper et al., 2008).

Degradation of Hyp by PRODH2/OH-POX initiates formation of several metabolites, including high-energy amino acid, Gly. The main source of Hyp is ECM collagen. This is particularly important energy substrate for cancer cells in conditions when glucose supply is limited. In such a case, cancer cells may select Pro and Hyp as an alternative energy source, since they have an advantage over fatty acids and glutamine (Gln), which like glucose require delivery by the circulation. Therefore, these amino acids may represent energy substrate. Utilization of these amino acids as an energy substrates is initiated by ECM collagen degradation by matrix metalloproteinases (MMPs) (Ii et al., 2006). Therefore, increase in the amount and activity of these enzymes favors tumor progression, metastasis and angiogenesis (Surazynski et al., 2008a; Surazynski et al., 2008b; Surazynski et al., 2005; Ii et al., 2006).

**FIGURE 4:**
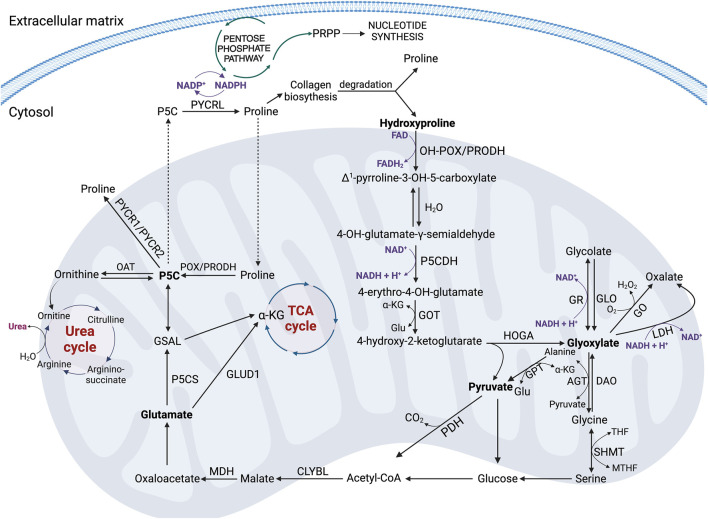
The key metabolic pathways of proline and hydroxyproline in the cell. Acetyl-CoA, acetyl coenzyme A; AGT, alanine-glyoxylate aminotransferase; CLYBL, Citramalyl-CoA lyase; DAO, D-amino oxidase; FAD, oxidized form of flavin adenine dinucleotide; FADH2, reduced form of flavin adenine dinucleotide; GLO, glycolate oxidase; Glu, glutamate; GLUD1, glutamate dehydrogenase 1; GO, glyoxylate oxidase; GOT, glutamate-OAA transaminase; GPT, glutamate pyruvate transaminase; GR, glyoxylate reductase; GSAL, L-glutamate-γ-semialdehyde; HOGA – 4-hydroxy-2-ketoglutarate aldolase; Hyp, hydroxyproline; α-KG, α-ketoglutarate; LDH, lactate dehydrogenase; MDH, malate dehydrogenase; MTHF, N5,N10-methylene- tetrahydrofolate; NAD+ – oxidized form of nicotinamide adenine dinucleotide; NADH, reduced form of nicotinamide adenine dinucleotide; NADP+, oxidized form of nicotinamide adenine dinucleotide phosphate; NADPH, reduced form of nicotinamide adenine dinucleotide phosphate; OAT, ornithine aminotransferase; OH-POX/PRODH2 – hydroxyproline oxidase/proline dehydrogenase 2; PDH, pyruvate dehydrogenase; POX/PRODH, proline oxidase/proline dehydrogenase; Pro, proline, PRPP, phosphoribosyl pyrophosphate; PYCR 1/2/L, P5C reductase 1/2/L; P5C, Δ1-pyrroline-5-carboxylate; P5CDH–P5C dehydrogenase; P5CS, P5C synthase; SHMT, serine hydroxymethyltransferase; THF, tetrahydrofolate.

Hyp oxidation dependently on the participating cofactor, can lead to different products. In the presence of FAD, OH-POX catalyzes transformation of Hyp into OH-P5C that further undergoes non-enzymatic hydrolysis to 4-hydroxy-glutamate-γ-semialdehyde (Moxley et al., 2011) and conversion to four-erythro-4-hydroxy-glutamate (4-OH-Glu) by P5CDH (Valle et al., 1979). Next, it is converted to ketoacid: 4-hydroxy-2-ketoglutarate (HOG) by glutamate-OAA transaminase (GOT) that is accompanied by the conversion of α-KG to Glu (Adams and Frank, 1980). The final step in these processes is the conversion of HOG to glyoxylate and pyruvate using the mitochondrial enzyme 4-hydroxy-2-ketoglutarate aldolase (HOGA) (Riedel et al., 2011; Goldstone and Adams, 1962; Giafi and Rumsby, 1998). Both transaminase and aldolase, which catalyze the reversible conversion of 4-hydroxy-glutamate to pyruvate and glyoxylate, are sterically nonselective for the configuration of the hydroxyl group (-OH) (Figure 4) (Summitt et al., 2015). Pyruvate is trans-aminated to alanine by the activity of glutamate pyruvate transaminase (GPT). This one can be converted to glucose or, with the involvement of pyruvate dehydrogenase (PDH), to Acetyl-CoA, which in reaction with the mitochondrial enzyme citramalyl-CoA lyase (CLYBL) contributes to the formation of malate. Malate, in turn, with the participation of malate dehydrogenase (MDH) and NAD^+^ as a cofactor, is converted to oxaloacetate, as a substrate for Glu. It is one of the key energy sources in the cell, the formation of which is a consequence of the transformations taking place in the Urea cycle (UC) or the transformation with the participation of glutamate dehydrogenase 1 (GLUD1) into α-KG, as a substrate for the TCA cycle (Figure 4) (Wu et al., 2019). A particularly interesting pathway of Glu metabolism is the reaction occurring under the influence of P5C synthase (P5CS), leading to the formation of P5C as a central component, which links the TCA cycle, UC, and Pro cycle (PC) to regulate the energy level in the cell. Formed from Glu, P5C can be converted to Orn by ornithine aminotransferase (OAT), providing a substrate for the UC (Figure 4) (Wu et al., 2019). Moreover, P5C metabolism in the cell is accompanied by a transfer of the reduction-oxidation potential, which affects the NADP^+^/NADPH ratio and a variety of processes, such as the synthesis of phosphoribosyl pyrophosphate (PRPP) and purine ribonucleotides, which are crucial for DNA synthesis (Yeh and Phang, 1983). Thus, Glu-P5C conversion is important, not only due to the regulation of the cell’s bioenergetics, but also both pro-survival and pro-apoptotic processes.

Glyoxylate, as the second product of HOGA activity besides pyruvate, can be reduced to glycolate by glyoxylate reductase (GR) accompanied by the conversion of NADH + H^+^ to NAD^+^ (Figure 4). This reaction occurs in the mitochondrion or shortly after entry to the cytoplasm (Giafi and Rumsby, 1998). Glycolate can then be converted to glyoxylate by glycolate oxidase (GLO), and glyoxylate in peroxisomes is converted to Gly by the activity of alanine-glyoxylate aminotransferase (AGT) (Danpure and Jennings, 1986). Gly can be a source of glyoxylate in a reverse reaction catalyzed by D-amino oxidase (DAO). It can function as a substrate for serine hydroxymethyltransferase (SHMT) and undergo conversion to serine, which then produces glucose, Acetyl-CoA and ATP in glycolysis (Figure 4). The fate of Acetyl-CoA and the metabolic consequences of its transformations are similar to those described above. They lead to the release of energy in the form of ATP.

There is also an alternative pathway of glyoxylate metabolism in the cell. Glyoxylate under the influence of glyoxylate oxidase (GO) or lactate dehydrogenase (LDH) is converted to oxalate. These reactions are accompanied by the conversion of O_2_ to H_2_O_2_ and NADH + H^+^ to NAD^+^, respectively (Figure 4) (Summitt et al., 2015; Wu et al., 2019).

In the context of regulation of energy in cancer cell particularly important is metabolism of Hyp into other amino acids, e.g., Gly, which gives high energy yield. The conversion of 1 mol of Hyp into 1 mol of Gly produces 4 mol of energy stored in ATP, while 1 mol of threonine and glucose, Glu or choline each produce 2.5 mol of Gly. Thus, the energy yield is 60% higher with Hyp compared to other substrates (Li and Wu, 2018).

## 3 The role of Hyp in transcriptional regulation

### 3.1 The role of Hyp in the regulation of HIF-1α transcriptional activity

**FIGURE 5:**
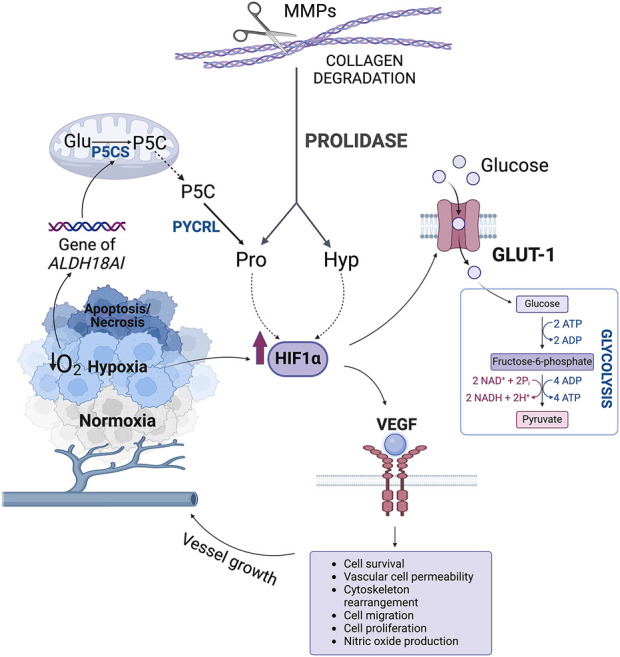
The role of hydroxyproline in the regulation of HIF-1α transcriptional activity. ADP, adenosine diphosphate; ATP, adenosine triphosphate; Glu, glutamate; GLUT-1, Glucose Transporter-1; HIF-1α, hypoxia-inducible factor 1α Hyp, hydroxyproline; MMPs, metalloproteinases; NAD+, oxidized form of nicotinamide adenine dinucleotide; NADH, reduced form of nicotinamide adenine dinucleotide; P5C, Δ1-pyrroline-5-carboxylate; P5CS, P5C synthase; Pro, proline; PYCRL, P5C reductase L; VEGF, Vascular Endothelial Growth Factor.

In proliferating cancer cells forming tumor, hypoxia develops as a result of reduced oxygen level. It contributes to upregulation of transcriptional activity of hypoxia-inducible factor 1α (HIF-1α) (Tang et al., 2018). In the presence of oxygen, Pro residues in the oxygen-dependent domain (ODD) of HIF-1α are hydroxylated. It determines HIF-1α proteasomal degradation (Surazynski et al., 2008a). In hypoxia, Pro residues of ODD domain in HIF-1α are not hydroxylated leading to the increase in HIF-1α nuclear level and transcriptional activity. Evidence was provided in human colorectal carcinoma cell line (RKO) in which a protein reporter plasmid was constructed that is a fusion product of the ODD fragment of HIF-1α with luciferase (CMV-Luc-ODD). ODD is a domain in which Pro-402/564 is hydroxylated. ODD hydroxylation is required for the interaction of HIF-1α with the Von Hippel-Lindau (VHL) tumor suppressor protein, key process for ubiquitination and proteasomal degradation of HIF-1α (Jaakkola et al., 2001; Mole et al., 2001). In the cited studies, the CMV-Luc-ODD reporter was transfected into the cells. There was a significant increase in Luc-ODD level in prolidase (PEPD)-transfected cells, indicating a reduction in fusion protein degradation. Under the effect of Gly-Pro or Gly-Hyp, there was also an increase in Luc-ODD level. This was especially pronounced by the effect of Gly-Hyp, at least in cells overexpressing PEPD (Surazynski et al., 2008a). Further studies have shown that Pro and Hyp stabilize HIF-1α through inhibition of its degradation (Surazynski et al., 2008a; Phang, 2022). Evidence was provided that overexpression of PEPD in RKO cells accompanied by increase in intracellular concentration of Pro and Hyp increased expression of nuclear HIF-1α and the expression of HIF-1-dependent gene products, such as Vascular Endothelial Growth Factor (VEGF) and Glucose Transporter-1 (Glut-1) (Figure 5) (Surazynski et al., 2008a). Moreover, differential expression of PEPD in two breast cancer cell lines, MCF-7 and MDA-MB-231, showed PEPD-dependent differences in HIF-1α levels (Surazynski et al., 2008a). It has been also documented that in RKO cells Hyp is more effective in upregulation of transcriptional activity of HIF-1α than Pro (Surazynski et al., 2008a). It could be particularly relevant in cancers characterized by elevated levels of PEPD expression, which contributes indirectly to increased expression and stabilization of HIF-1α in response to hypoxia, which in turn is crucial in promoting angiogenesis, glycolysis, metastasis and survival (Wu et al., 2019).

In addition to HIF-1α, other transcription factors such as p53, MYC, and the signaling pathways regulated by protein kinase B (PKB, known also as AKT), and PI3K are involved in the regulation of Pro metabolism during oncogenic transformation (Liu et al., 2015). p53, a key tumor suppressor, activates PRODH/POX and PRODH2/OH-POX, enzymes essential for Pro metabolism (Phang and Liu, 2012; D’Aniello et al., 2020; Geng et al., 2021). The activation of these enzymes by p53 suggests that Pro and Hyp metabolism is intricately linked to the cell’s response to stress and its ability to maintain metabolic homeostasis (Phang, 2019). This activation can shift cellular metabolism to support growth and survival, even in the face of DNA damage or oncogenic stress. MYC, AKT, and PI3K, all of which are implicated in the regulation of Pro synthesis, further contribute to the metabolic reprogramming that is characteristic for cancer cells (Liu et al., 2012; Phang, 2019). These pathways are responsible for the enhanced synthesis of Pro, facilitating tumorigenic processes by supporting cellular proliferation and survival.

Pro hydroxylation in proteins may also influence other post-translational modifications. For instance, hydroxylation of Pro residues in AKT, as well as in tumour suppressor p53 or eukaryotic elongation factor 2 (DYRK1A/B) were found to play important role in regulation of the protein’s phosphorylation and cell signalling (Zurlo et al., 2016; Hu et al., 2022). An example is PKB/AKT that when hydroxylated binds directly to VHL inhibiting PKB/AKT phosphorylation, leading to decrease in cancer cell proliferation. In contrast, hydroxylation of p53 induces phosphorylation of the protein and tumour suppressing activity (Zurlo et al., 2016; Hu et al., 2022). Thus, the regulation of transcription factors such as HIF-1α, p53, MYC, and the activation of signaling pathways like AKT and PI3K, is tightly coupled with Pro and Hyp metabolism. These pathways not only influence cellular processes essential for tumor growth, such as angiogenesis and glycolysis, but also contribute to the epigenetic reprogramming of cancer cells (D’Aniello et al., 2020; Wicks and Semenza, 2022). By modulating the activity of enzymes that control histone and DNA methylation, Pro metabolism creates a link between cellular metabolism and the regulation of gene expression. This integrated approach to cellular regulation underscores the complexity of cancer biology, where metabolic changes drive epigenetic modifications that enable the progression and adaptation of tumor cells in hostile environments.

### 3.2 The role of Hyp in epigenetic regulation

The induction of PRODH/POX and PRODH2/OH-POX by p53 and activation of Pro synthetic enzymes by MYC oncogene, AKT and PI3K suggests that the Pro and Hyp metabolism may play an important role in metabolic reprogramming during oncogenic transformation (Phang, 2019). In fact, increased expression of PYCR1 is associated with many human cancers (D'Aniello et al., 2020), while knockdown of PYCR1 in cancer cells transplanted into mice markedly decreased tumor growth. Interestingly, the availability of Pro does not mitigate the inability to synthesize Pro suggesting the specific mechanism for compartmentation of endogenously synthesized Pro, Pro-derived from collagen degradation and Pro available for the synthesis of collagen. The potential mechanism for routing of synthesized Pro to collagen synthesis and the metabolic role of collagen turnover has been recently described (D'Aniello et al., 2020). Furthermore, the mechano-regulatory properties of collagen and the role of Kindlin-2 and Pinch-1, two sensors of collagen stiffness caused upregulation of PYCR1 strongly suggesting that Pro synthesis is linked to a functional role of collagen (Chen et al., 2021). These regulatory functions of Pro and collagen metabolism in influencing metabolic epigenetics has been reviewed (D'Aniello et al., 2020; D'Aniello et al., 2015). Several lines of evidence suggest that prolyl-4-hydroxylases play critical role in epigenetic regulation. They belong to the family of dioxygenases that include collagen prolyl hydroxylases (P4HA1, P4HA2), prolyl hydroxylase of DNA and histone demethylases and HIF-1α prolyl hydroxylase (D’Aniello et al., 2019). They all are Fe^+2^, ascorbate and α-KG-dependent enzymes. Since collagen prolyl hydroxylases compete with DNA and histone demethylases for ascorbate and α-KG, the rate of collagen biosynthesis, which is dependent on hydroxylation of prolyl residues, influences metabolic epigenetics. Upregulation of collagen prolyl hydroxylase and its exhaustion of ascorbate and α-KG may compete with DNA and histone demethylases (that require the same cofactors) to influence metabolic epigenetics. In example, overexpression of P4HA2 has been implicated in development of several cancers (Jarzą; BENDER et al., 2005; Jiang et al., 2018; D’Aniello et al., 2019; Li et al., 2019; Lin et al., 2020) including breast cancer (Toss et al., 2018). Conversely, depletion of P4HA2 inhibited breast cancer cell proliferation and invasiveness *in vitro* and *in vivo*, by reducing collagen deposition (Xiong et al., 2014). Similarly, elevated P4HA1 expression was described in pancreatic ductal adenocarcinoma (Cao et al., 2019), head and neck squamous cell carcinoma (Li et al., 2020), oral squamous cell carcinoma (Kappler et al., 2017), and prostate cancer (Chakravarthi et al., 2014).

Since Pro biosynthesis is rate limiting for the incorporation of Pro into collagen, the rate of Pro hydroxylation is also influenced. Thus, synthesized Pro can influence the rate of collagen synthesis with hydroxylation of Pro residues and provide the metabolic linkage to epigenetic regulation.

## 4 The role of Hyp in apoptosis and tumor progression

Collagen prolyl 4-hydroxylase is overexpressed in several cancer cells, while silencing this enzyme inhibits tumorigenesis *in vivo* (Shi et al., 2021). Despite the role of prolyl 4-hydroxylases in epigenetic regulation, they contribute to increase in cellular level of Hyp, as a result of collagen degradation. The final step of collagen degradation is catalysed by PEPD. An increase in the enzyme expression and activity was demonstrated to correlate with occurrence of several cancers (Wilk et al., 2021). The pro-survival activity of PEPD in cancer cells was explained by ability of PEPD to bind and inactivate p53, upregulate HIF-1α, AKT, p-38 and increase in Hyp cellular level (Wilk et al., 2021). Therefore, Hyp metabolism in cancer cells is of considerable interest.

PRODH2/OH-POX is an enzyme under transcriptional control of p53 (Cooper et al., 2008; Donald et al., 2001; Polyak et al., 1997; Maxwell and Davis, 2000) therefore, the involvement of Hyp and PRODH2/OH-POX in the regulation of cell death is of particular interest for potential anticancer therapy (Cooper et al., 2008). The product of degradation of Hyp by PRODH2/OH-POX, OH-P5C could be also converted by OH-P5C reductase to recycle NADP^+^ in PPP, yielding nucleotides for DNA synthesis. It is also of interest that Hyp is the most efficient energy producing amino acid that yields Gly through multistep catalytic processes described in previous chapter (Wu et al., 2019).

**FIGURE 6:**
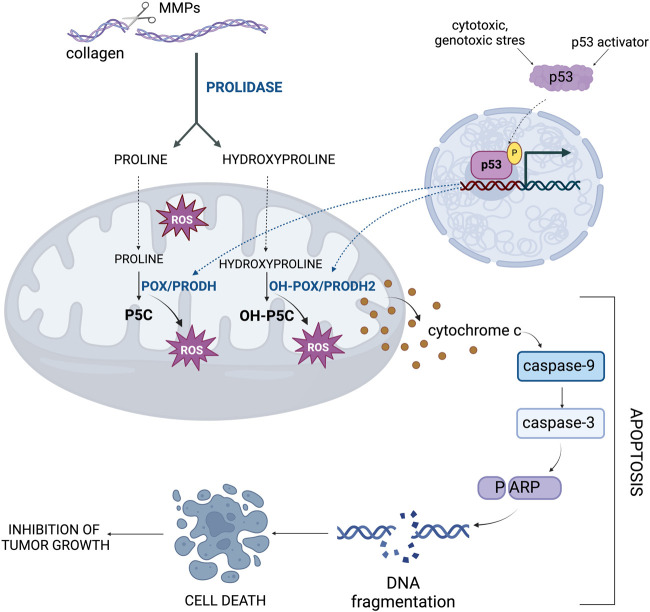
The importance of hydroxyproline metabolism in the regulation of apoptosis. The gene encoding OH-POX/PRODH2, the enzyme that degrades Hyp, is under the transcriptional control of p53 as a key activator of apoptosis. MMPs, metalloproteinases; OH-POX/PRODH2 - hydroxyproline oxidase/proline dehydrogenase 2; OH-P5C, Δ^1^-pyrroline-3-OH-5-carboxylic acid; POX/PRODH, proline oxidase/proline dehydrogenase; P5C, Δ^1^-pyrroline-5-carboxylate; ROS, reactive oxygen species.

Hyp, similarly as Pro converted to P5C by PRODH/POX, is transformed to OH-P5C by PRODH2/OH-POX. This reaction is accompanied by generation of ROS, which predisposes to the induction of apoptosis (Figure 6) (Liu et al., 2021; Kononczuk et al., 2015). The preference of Hyp for PRODH2/OH-POX is due to the characteristic structure of the enzyme, in which residues 157–515 contain a catalytic core with one FAD molecule (Summitt et al., 2015). It was confirmed by kinetic analyses and the determination of a *K*_*cat*_/*K*_*m*_ value of 0.93 M-1-s-1, which is 12 fold higher for Hyp compared to Pro (Summitt et al., 2015).

The mechanism for POX or OH-POX-dependent apoptosis is well established (Kazberuk et al., 2022; Liu et al., 2006; Hu et al., 2007; Liu et al., 2021; Liu et al., 2005; Maxwell and Rivera, 2003). POX/OH-POX catalyzes reactions (conversion of Pro to P5C and Hyp to OH-P5C), during which ROS are formed inducing apoptosis. It has been documented in RKO and LoVo cell lines expressing native-type of p53 (Cooper et al., 2008). Treatment of these cells with Doxorubicin, an activator of p53 protein contributed to increase in PRODH2/OH-POX expression and activity. In contrast, in Doxorubicin treated colorectal adenocarcinoma HT29 and HCT15 cell lines expressing mutated type of p53 this phenomenon did not occur. These findings suggest that PRODH2/OH-POX-induced apoptosis is a p53-dependent process (Figure 6). Transfection of the cells with siRNA for PRODH2/OH-POX showed a significant decrease in PRODH2/OH-POX expression and catalytic activity, resulting in a reduction in the amount of ROS generated by conversion of Hyp into OH-P5C. This demonstrates that PRODH2/OH-POX is involved in ROS formation. A similar effect was observed in cells transfected with siRNA for p53, confirming functional dependence of PRODH2/OH-POX on p53 expression (Cooper et al., 2008).

Interestingly, a non-physiological isomer of Hyp, cis-4-hydroxyproline (CHP) induces caspase-independent apoptosis. In pancreatic cancer cell line (DSL6A) CHP activates intracellular proteolytic processes, including caspase-independent degradation of Focal Adhesion Kinase (FAK) inducing a loss of cancer cell adhesion. It is accompanied by CHP-induced *endoplasmic reticulum* (ER) stress cascade and an increase in the expression of GRP78 and GADD153. Prolonged exposure of DSL6A cells to CHP induces apoptosis (Mueller et al., 2006).

## 5 Potential therapeutic implications

**FIGURE 7:**
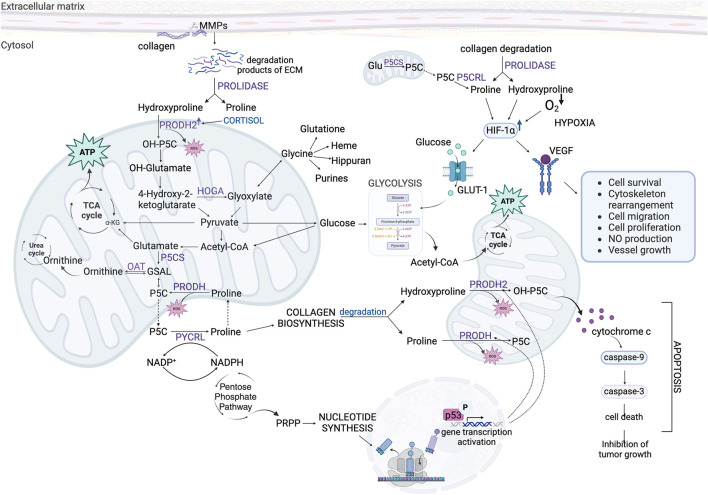
The role of Hydroxyproline and Proline in complex metabolic regulations affecting survival/apoptosis in cancer cells. Acetyl-CoA, acetyl coenzyme A; ATP, adenosine triphosphate; ECM, extracellular matrix; Glu, glutamate; GLUT-1, Glucose Transporter-1; GSAL, L-glutamate-γ-semialdehyde; HIF-1α, hypoxia-inducible factor 1α; HOGA – 4-hydroxy-2-ketoglutarate aldolase; MMPs, metalloproteinases; NADP^+^, oxidized form of nicotinamide adenine dinucleotide phosphate; NADPH, reduced form of nicotinamide adenine dinucleotide phosphate; OAT, ornithine aminotransferase; OH-P5C, Δ1-pyrroline-3-OH-5-carboxylic acid; OH-α-KG, α-hydroxyketoglutarate; PRODH, proline dehydrogenase; PRODH2, proline dehydrogenase 2; PRPP, phosphoribosyl pyrophosphate; P5C, Δ1-pyrroline-5-carboxylate; P5CS, P5C synthase; PYCRL, P5C reductase L; TCA cycle, tricarboxylic acid cycle; VEGF, Vascular Endothelial Growth Factor; α-KG, α-ketoglutarate.

Recent studies cast new light on the role of Hyp in cancer cell metabolism. Hyp is involved in energy and redox homeostasis, DNA synthesis, activation of some transcription factors and epigenetic regulation. These activities are of great importance in regulation of apoptosis/survival, angiogenesis and metastasis. The data presented in this review suggest that increase in cellular level of Hyp contribute to pro-survival phenotype of cancer cells (Figure 7). It suggests that Hyp metabolism could be considered as a target in novel strategy for cancer treatment. Developing stimulators of PRODH2/OH-POX catalytic activity could induce ROS-dependent apoptosis in cancer cells. Moreover, increase in Hyp degradation by PRODH2/OH-POX contributes to decrease in cellular level of Hyp, eliminating highly energetic amino acid which supplements the energy needs of cancer cells. Decrease in Hyp level promotes also HIF-1α degradation eliminating this transcription factor as a tumor growth agent. Therefore, upregulation of Hyp degradation in cancer cells could affect energy level, redox balance, proliferative processes and induce apoptosis. This strategy could be particularly important because Hyp is not formed *de novo* in the cell but comes from degradation of proteins, mainly collagen (Gorres and Raines, 2010).
